# Ubiquitous Water-Soluble Molecules in Aquatic Plant Exudates Determine Specific Insect Attraction

**DOI:** 10.1371/journal.pone.0003350

**Published:** 2008-10-08

**Authors:** Julien Sérandour, Stéphane Reynaud, John Willison, Joëlle Patouraux, Thierry Gaude, Patrick Ravanel, Guy Lempérière, Muriel Raveton

**Affiliations:** 1 Laboratoire d'Ecologie Alpine, Equipe Perturbation Environnementale et Xénobiotiques, UMR CNRS n°5553, Université Joseph Fourier, BP 53, Grenoble, France; 2 Laboratoire de Chimie et Biologie des Métaux, UMR 5249 UJF/CEA/CNRS, Institut de Recherches et Technologies en Sciences du Vivant, CEA-Grenoble, Grenoble, France; University of Oxford, United Kingdom

## Abstract

Plants produce semio-chemicals that directly influence insect attraction and/or repulsion. Generally, this attraction is closely associated with herbivory and has been studied mainly under atmospheric conditions. On the other hand, the relationship between aquatic plants and insects has been little studied. To determine whether the roots of aquatic macrophytes release attractive chemical mixtures into the water, we studied the behaviour of mosquito larvae using olfactory experiments with root exudates. After testing the attraction on *Culex* and *Aedes* mosquito larvae, we chose to work with *Coquillettidia* species, which have a complex behaviour in nature and need to be attached to plant roots in order to obtain oxygen. This relationship is non-destructive and can be described as commensal behaviour. Commonly found compounds seemed to be involved in insect attraction since root exudates from different plants were all attractive. Moreover, chemical analysis allowed us to identify a certain number of commonly found, highly water-soluble, low-molecular-weight compounds, several of which (glycerol, uracil, thymine, uridine, thymidine) were able to induce attraction when tested individually but at concentrations substantially higher than those found in nature. However, our principal findings demonstrated that these compounds appeared to act synergistically, since a mixture of these five compounds attracted larvae at natural concentrations (0.7 nM glycerol, <0.5 nM uracil, 0.6 nM thymine, 2.8 nM uridine, 86 nM thymidine), much lower than those found for each compound tested individually. These results provide strong evidence that a mixture of polyols (glycerol), pyrimidines (uracil, thymine), and nucleosides (uridine, thymidine) functions as an efficient attractive signal in nature for *Coquillettidia* larvae. We therefore show for the first time, that such commonly found compounds may play an important role in plant-insect relationships in aquatic eco-systems.

## Introduction

Plant attractiveness to insects has been widely studied in plant-herbivore-parasitoid interactions. Volatile Organic Compounds (VOCs) emitted by plant–herbivore interactions are of importance for host or prey location by parasitoids and predators of phytophagous insects [1]–[6]. Some plants are able to release volatile infochemicals during an attack by specific herbivorous insects that attract predators specialized on the herbivore species. These predators respond to these chemical signals by attacking the herbivores, thereby reducing the plant's tissue loss by herbivory [7]–[8]. More than 1000 VOCs are involved in such interactions [9], and in a single plant–herbivore complex 30–50 VOCs are frequently detected by chromatographic (GC-MS) analysis [6]. Among the wide variety of attractive compounds for terrestrial insects, the majority are represented by species-specific chemicals, mainly produced by plant secondary metabolism, such as polyphenols, isothiocyanates, terpenoids, fatty acid derivatives, benzoids and nitrogen or sulfur containing compounds [9], [10]–[11]. Ubiquitous metabolites such as alcohols or sugars have been shown to be involved in such interactions, but it seems that they act as phagostimulants [12]. It has been suggested that not only is the composition of the plant signal important in the insect attractiveness by plant, but also the proportion of the different VOCs presents in the emitting signal [13]. It has been demonstrated that plants emit distinct volatile blends in response to two closely related herbivore species, and that the parasitoids are able to distinguish these two signals suggesting a sophisticated chemical system of plant-herbivore-parasitoid interaction [14].

In aquatic systems, VOCs dissolved in water may be responsible for air insect attraction [15], [16]. Nevertheless, non-volatile chemicals (VOCs-like), which typically have low mobility in air, may become mobile in water and might play a role in the attraction of water-living insects. In total aquatic system, studies of animal responses to chemical stimuli in aquatic systems have focused primarily on fish and non-insect invertebrates with a view to elucidating the mechanisms of chemical communication in a predator-prey relationships. In the majority of these studies, it was demonstrated that the compounds implicated in the attraction were feeding stimulants such as glycine, amino acids, sugars or organic acids [17], [18]. Few studies have examined the orientation of herbivores to plant extracts or plant-conditioned water but all of them concerned sea water animals (i.e. sea urchins [19], [20] and estuarine snails [21]). Only one study concerning a plant-insect interaction in freshwater has been described [22]. In this study the authors demonstrated that the milfoil weevil (*Euhrychiopsis lecontei* Dietz, 1896; Coleoptera: Curculionidae) was attracted by chemicals released by an invasive host-plant (*Myriophyllum spicatum* Linnaeus, 1753; Haloragaceae) for feeding, ovipositing and mating. Host plant attractions often involve a mixture of VOCs-like, and the effective concentration of one attractant can be modified, when diluted in a specific mixture [23], [24]. However, this phenomenon was not observed by Marko et al. [22], who detected no synergism between attractants in the *E. lecontei-M. spicatum* relationship.

There is public health concern about *Coquillettidia* mosquitoes (Diptera: Culicidae) as they are potential bridge vectors of West Nile virus [25]–[27], Eastern Equine Encephalomyelitis [28] and *Dirofilaria* nematodes [29], and are widely distributed geographically. The colonization patterns of emergent aquatic macrophytes by the larval stages of the mosquito *Coquillettidia* (*Coquillettidia richiardii* Ficalbi, 1889 and *Coquilletidia buxtoni* Edwards, 1923) are of special interest because they remain attached to host-plants in deep nutrient-rich and hypoxic aquatic environments. This plant–insect interaction appears to be regulated by the need of the continuously submerged larvae to find oxygen in the aerenchymal channels of roots [30]. Such a plant–insect relationship seems to be non-destructive for the host plant and therefore could be described as a commensalist interaction. Our previous studies have demonstrated that *C. richiardii* larvae are disturbed by the presence of light, since they are lucifugous and their attachment to plant roots decreased with light intensity [31]. Moreover, the absence of oxygen appears to initiate the search of larvae for host-plant roots to attach to.

In the present study, we examined the attraction of *Coquillettidia* larvae by plant root exudates from different host and non-host species, with the objective to identifying the chemical compounds involved in the attraction. Chemical profiles of root exudates were analysed and the larval attraction of individual compounds was determined, five compounds responsible for *Coquillettidia* larvae attraction by plant were identified and their optimal attractive concentrations were measured. Finally, the behavioural responses of larvae to different chemical mixtures were determined to investigate potential attraction by synergism. Our results suggest that attraction of *Coquillettidia* larvae to the host plant is mediated synergistically by a mixture of simple, water-soluble compounds released by the roots.

## Results

### Multiple plant root exudates reveal a common attraction for *Coquillettidia* larvae

*Coquillettidia* behaviour was tested initially by measuring attraction in a four-channel olfactometer, as described in Fig. S1.

**Figure 1:**
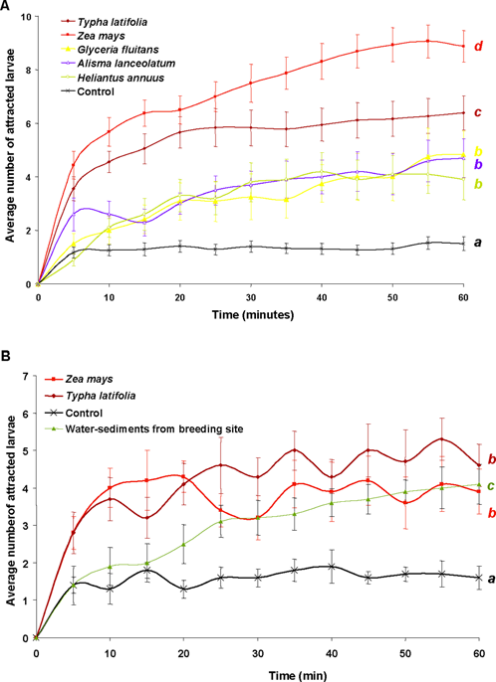
Responses of *Coquillettidia* larvae to different plant exudates. 1A, attractiveness measured with host and non-host plants. Roots of plants were put in 4-channel-olfactometer with a water flow rate of 3.2 ml/min. 1B, attractiveness to the root exudates solution of host plants (*Maize* and *Typha*). Responses are expressed in Average (±SE) number of attracted larvae in function of time, under red light. ANOVA, p<0.0001, Superscripts*^a,b,c,d^* indicate differences at a significant level of 0.05.

Different plant species were tested for attraction, including the following Monocotyledones: natural aquatic host-plant (the cattail: *Typha latifolia* Linnaeus, 1753), aquatic non-host-plants (*Alisma lanceolatum* Withering, 1796; *Glyceria fluitans* Linnaeus, 1810) and non-aquatic cultivated plant (the maize: *Zea mays* Linnaeus, 1753); and Dicotyledones: non-aquatic plant (*Helianthus annuus* Linnaeus, 1753). These six plant species elicited a significant positive attraction with a better larval response towards maize and cattail (Fig. 1A). Such results indicated that the compounds implicated in larvae attraction might be commonly found products exudated by roots. To test this hypothesis, additional experiments were performed using root exudate solutions obtained from 1-day hydroponic cultivation of the two most attractive plants: *T. latifolia*, a natural host and *Z. mays*, a non-natural host (Fig. 1B). *Coquillettidia* larvae were significantly attracted by maize (ANOVA, *F*_1.23_ = 6.11, p<0.0001) and cattail exudates (ANOVA, *F*_1.23_ = 12.81, p<0.0001). Moreover, water samples originated from breeding sites (sediments were collected nearby roots and centrifuged to extract water) were significantly attractant for *Coquillettidia* larvae (ANOVA, p<0.0001). No difference between *Z. mays* and *T. latifolia* exudate responses was observed (*F*_1.23_ = 1.15, p = 0.3) suggesting that *Coquillettidia* larvae were attracted by common natural compounds released by cell root in the surrounding water.

In order to test the specificity of *Coquillettidia* larvae attraction by plant roots, the behaviour of two other mosquito species was studied (*Culex pipiens pipiens* Linnaeus, 1758 and *Aedes stegomyia aegypti* Linnaeus, 1762). These species revealed a very different behaviour since their larvae were not significantly attracted by plant roots contrary to *Coquillettidia* larvae which showed a positive response (ANOVA, p<0.0001).

### Root compounds involved in *Coquillettidia* larvae attraction

Root exudate compounds identified by GC-MS analyses are listed in Table 1. Most of these products corresponded to amino acids, organic acids, sugars, pyrimidines, nucleosides, fatty acids and glycerol, in accordance with Kumar et al. [32]. Each compound was tested at two different concentrations on *Coquillettidia* larvae in order to establish its attraction potentiality using a four-channel olfactometer (Fig. S1). Molecules from the amino acid and organic acid groups were not attractive for mosquito larvae. Neither pentitol, nor three other sugars, ribose, deoxyribose and glucose (described in maize root exudates by Kamilova et al. [33]) were attractive. Fatty acids detected at a high level under natural conditions were not attractive for larvae, nor were secondary metabolites that were not detected in our samples but have been previously described in root exudates, such as rutine [34], quercetine [35] and DIMBOA [36]. On the other hand, pyrimidines, nucleosides and glycerol (Table 1) were attractive.

**Table 1:** Compounds detected in plant root exudates (*Zea mays*, *Typha latifolia* and in water-sediments from natural ponds) and their potential attractiveness on *Coquillettidia* larvae.

Compounds		Detection			Attraction	
		*t_R_* a (min)	Exudates	*In natura*	100 µM	1000 µM
**Amino acids**	Threonine	7.72	*Zea*	Yes	No	No
	Proline	7.48	*Typha*	ND	No	No
	Serine	7.64	ND	Yes	No	No##
**Organic acids**	Ferrulic	10.47	*Zea*	ND	No	No
	Coumaric	9.63	*Zea*	ND	No	No
**Sugars**	Pentitol	8.14	*Zea*	ND	No	No
**Purines**	Uracile	7.82	*Zea/Typha*	Yes	Yes***	Yes***
	Thymine	7.79	*Zea/Typha*	Yes	Yes***	Yes***
	Cytosine	8.19	*Zea/Typha*	Yes	No	No
**Nucleosides**	Uridine	14.32	*Zea/Typha*	Yes	Yes***	Yes**
	Thymidine	13.89	*Zea/Typha*	Yes	No	Yes**
**Fatty acids**	Monopalmitin	17.31	*Zea/Typha*	Yes	No^b^	/b
**Polyols**	Glycerol	7.33	*Zea/Typha*	Yes	Yes	Yes***

a Retention time corresponding to the silylated compound forms.

b Water limit solubility = 10 µM.

ND, Not Detected.

ANOVA.

*** p<0.001.

** p<0.01.

* p<0.05.

## p<0.01.

**Figure 2:**
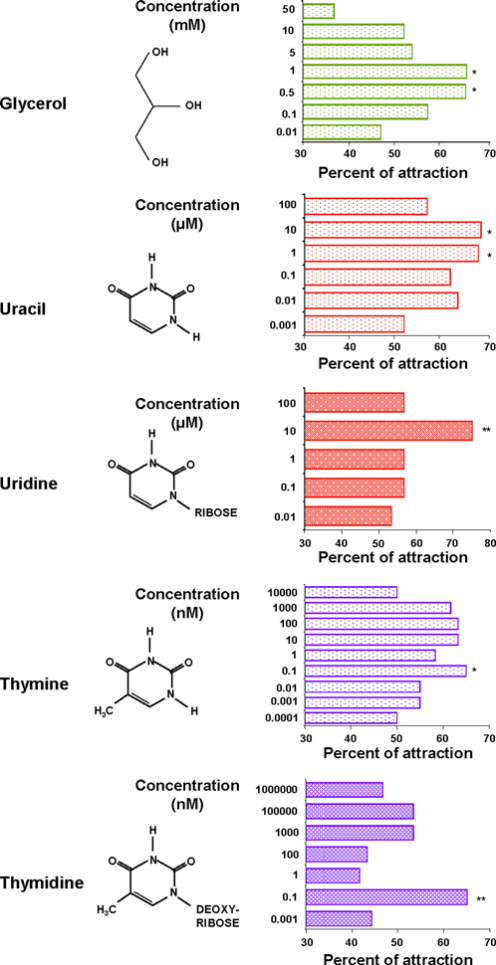
Responses of *Coquillettidia* in two choice-olfactometer to different compounds: glycerol, uracil, uridine, thymine and thymidine. χ^2^ test for significant differences between compound concentrations attractiveness. **, p<0.01; *, p<0.05. At least 80 larvae have made a choice in each experiment.

Therefore, to determine more precisely the level of attraction, *Coquillettidia* behaviour was tested using a two-choice olfactometer (Fig. S2). Larvae were attracted by pyrimidines such as uracil (2-oxo-4-oxo-pyrimidine) and thymine (5-methyl-uracil), which are two chemically similar molecules. First choice attraction experiments demonstrated that larvae were significantly attracted by uracil at 1 µM (χ^2^ = 4.03; p = 0.045) and 10 µM (χ^2^ = 4.17; p = 0.041) concentrations (Fig. 2). The percentage attraction corresponded respectively to 67.7 and 68.3 %. The attraction level for thymine was much lower since larvae were attracted at 0.1 nM concentration: 68% first choice at 0.1 nM (χ^2^ = 5.63; p = 0.0177), compared to about 63% for concentrations between 10 and 100 nM (χ^2^ = 2.97; p>0.05). Cytosine (2-oxo-4-aminopyrimide), which differs from uracil by an amino group (-NH_2_) in place of a carbonyl group (-C = O), was not attractive for mosquito larvae (data not shown).

Nucleosides corresponding to the attachment of sugars to the two abovementioned pyrimidines were both attractive for *Coquillettidia* larvae. The sugar moieties (ribose and deoxyribose) did not seem to play a role in the attraction of larvae since the responses observed with the nucleosides (uridine and thymidine, Table 1) were similar to those obtained with the corresponding pyrimidines (Fig. 2). First choice experiments demonstrated that uridine was significantly attractive at 10 µM (χ^2^ = 8.00; p = 0.005) with 75% of attraction whereas thymidine attracted larvae at a concentration of 0.1 nM with 65.4% of first choice (χ^2^ = 3.93; p = 0.048).

Among the compounds listed in Table 1, glycerol showed attractiveness but at much higher concentrations than the other compounds. First choice experiments (Fig. 2) demonstrated that *Coquillettidia* larvae were attracted by glycerol concentrations between 0.5 mM (65% first choice, χ^2^ = 3.93; p<0.048) and 1 mM (67% first choice, χ^2^ = 5.74; p = 0.0166).

### Synergy of *Coquillettidia* larvae attraction by root compounds

GC-MS analyses of root exudates and sediment samples showed that the concentrations of glycerol, uracil, thymine, uridine and thymidine were very similar in maize and cattail exudates (Table 2), although the glycerol concentration was ten times lower in cattail root exudates. On the other hand, the production rate of these compounds appeared to be higher in cattail than in maize, by a factor of 5, 4, 2, 3 and 3, respectively, for glycerol, uracil, thymine, uridine, thymidine.

**Table 2:** Relative amounts of compounds released from root plants of *Zea mays* and *Typha latifolia*.

Compounds	*Zea* exudates		*Typha* exudates		Sediment water
	Concentration (nM)	Rate^b^ (nmol/day/plant)	Concentration (nM)	Rate^b^ (nmol/day/plant)	Concentration (nM)
**Glycerol**	12	0.27	1.35	0.108	0.73
**Uracil**	<0.5^a^	<0.011	<0.5^a^	<0.04	<0.5^a^
**Thymine**	0.08	0.0018	0.04	0.003	6.63
**Uridine**	10	0.225	7.5	0.6	2.85
**Thymidine**	122	2.745	113	9.04	86.23

a Uracil was detected by MS-spectra analyses but quantities were under limit detection.

b Rate calculation: [Concentration (nM)×Volume (L)]/Number of days/Number of plants.

**Figure 3:**
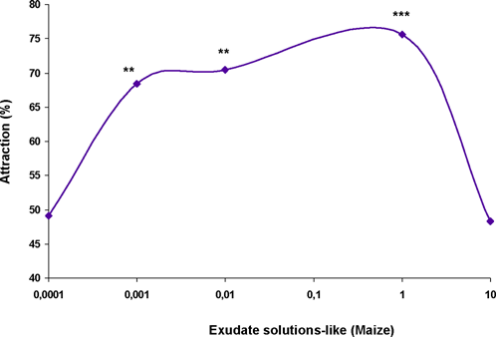
Responses of *Coquillettidia* in two choice-olfactometer to the mixture mimicking *Zea mays* and *Typha latifolia* root exudates. χ^2^ test for significant differences between compound concentrations attractiveness. ***, p<0.001; **, p<0.01. At least 80 larvae have made a choice in each experiment.

Based on these measurements, solutions mimicking exudates were prepared using pure chemicals and tap water. Experiments were performed using the two-choice olfactometer to measure first choice attractions (Fig. 3). *Coquillettidia* larvae were attracted by these mixtures and the response was amplified since the degree of first choice response was higher than response obtained for single compounds tests: 76.19% first choice for solutions mimicking cattail exudates (χ^2^ = 9.34; p = 0.002) and 75.64% first choice for solutions mimicking maize exudates (χ^2^ = 10.98; p = 0.0009). The attraction was maintained when the mixture was diluted a 100-fold (70%, χ^2^ = 6.22; p = 0.126) and 1000-fold (68%, χ^2^ = 5.34; p = 0.0209), whereas a mixture diluted 10000-fold induced a loss of attraction (49%, χ^2^ = 0.009; p = 0.926). On the other hand, a 10-fold concentrated mixture did not show attraction (48%, χ^2^ = 0.034; p = 0.853) and larval mobility was disturbed (10.6% did not show mobility).

## Discussion

Our initial working hypothesis was that the relationship between *Coquillettidia* and aquatic macrophytes, which is unusual among plant-insect interactions since it is non-destructive [37]–[39], was mediated by plant release of very specific compounds. However, our study clearly demonstrates that widespread plant molecules, at very low concentrations, were responsible for this interaction. For the first time in an aquatic environment, we demonstrate that a synergism between the different attractive compounds present in the emitted signal occurred in this particular plant–insect interaction.

*Coquillettidia* larvae were strongly attracted by plants while larvae of other species (*Aedes aegypti*, *Culex pipiens*) were indifferent towards all plants tested. Therefore, the plant-*Coquillettidia* interaction, which is necessary to the survival of larvae (O_2_ intake *via* plant aerenchymes for respiration [40]), was thought to be specific due to the release of VOCs-like compounds into the rhizosphere. However, despite decades of research, the identity of these aquatic VOCs-like compounds is still unclear [18]. Thus, in our first experiments we tested several molecules linked to plant secondary metabolism, but none of the flavonoids and hydroxamates (potentially present in monocotyledons) tested seemed to be involved in *Coquillettidia* larvae orientation. However, since many different plant species, such as *Typha latifolia*, *Alisma lanceolatum*, *Glyceria fluitans, Zea mays* and *Helianthus annuu*s, elicited a significant positive attraction, we have suggested that simple organic compounds commonly found in root exudates, might be responsible for the attraction observed. Non-volatile chemicals, which typically have low mobility in air may become mobile in water. The dynamics of plant–insect interactions are likely to be similar in aquatic and terrestrial systems, but the mechanism of interaction and the chemicals involved in these interactions may differ [22]. In aquatic systems, studies of animal responses to chemical stimuli have focused primarily on fish and non-insect invertebrates. In the majority of these studies it has been demonstrated that the compounds involved in the prey-predator interaction were feeding stimulants, such as glycine, amino acids, sugars and organic acids [17], [18]. In the present study, a large number of non-volatile molecules found in root exudates (organic acids, amino acids, nucleosides, sugars) were tested at various concentrations, but only a few were found to induce a behavioural attraction in *Coquillettidia* larvae. Our results led to the identification of glycerol, pyrimidines (uracil and thymine) and nucleosides (uridine and thymidine), as being responsible for the larval attraction. Glycerol and uracil have been previously described as attractants for the herbivore Coleoptera *Euhrychiopsis lecontei*, [22] with similar optimal concentrations (0.5–1 mM and 1–10 µM, respectively). On the other hand, thymine and thymidine, which were attractive at very low concentrations (0.1 nM), and uridine, which was attractive at 10 µM, have not previously been described as attractants. Glycerol was attractive at very high concentrations (0.5 to 1 mM) compared to the other compounds and lower concentrations did not induce a positive larval response. Even higher glycerol concentrations (10 to 50 mM), seemed to saturate the olfactory system of larvae, which became static and did not try to attach to a support. With regards to its such high effective concentrations and its chemical family, this particular compound could be considered as a food attractant for *Coquillettidia* larvae [12].

Glycerol is a common metabolite that can protect plant against abiotic stress [22]. The concentration range for attraction of *Coquillettidia* was similar to that for *Euhrychiopsis lecontei* in an aquatic system or for the attraction of by carbohydrates insects in terrestrial systems [11], [22]. Pyrimidines and pyrimidine nucleosides were attractive at much lower concentrations, between 0.1 and 1000 nM. This concentration range is more indicative of a plant-specific attractant that a nutrient [22]. Pyrimidines and pyrimidine nucleosides are ubiquitous compounds in plants and both molecules were found in broadleaf cattail and maize root exudates. However, the presence of these compounds in plant exudates raised the question as to why a plant would release expensive metabolites. In plant cells it has been shown that uridine is the second most common nucleoside after adenosine [22]. It has been suggested that the presence of uracil in exudates might be a function of its abundance and ubiquity in rapidly growing cells such as myristematic zones, where pyrimidine salvage was needed to support the high demand for nucleotides [41]. Indeed, the presence of uracil, uridine, thymine and thymidine in root exudates might be due to root cell death rather than active exudation [32].

The root exudates were constituted by a mixture of several compounds, including the five attractive molecules as demonstrated by the analysis of sediment water (0.7 nM glycerol, <0.5 nM uracil, 0.6 nM thymine, 2.8 nM uridine, 86 nM thymidine), associated to gases emanations (CO_2_, CH_4_) [32]. Our results demonstrated that *Coquillettidia* larvae were particularly sensitive to the mixture of these five compounds while larvae from other mosquito species (*Aedes* and *Culex* spp.) were not attracted. The attraction was amplified with 75% of positive responses and thus with concentrations 1,000- to 1,000,000-fold lower than those estimated with single compounds. At optimal concentrations, the mixture induced larval orientation to roots where larvae were able to perforate plant tissue and to attach on by the spiracular apparatus located in the respiratory siphon [40]. Then, it was possible to consider that these compounds induced larval attraction by a synergistic process. For the first time in aquatic environment, we have made the demonstration that a synergism between the different attractive chemicals contained in the emitted signal occurred in this particular plant insect interaction. This result was specific to *Coquillettidia* larvae because the mixture of glycerol and uracil did not induce an increase of the attractive response for *E. lecontei* [22].

**Figure 4:**
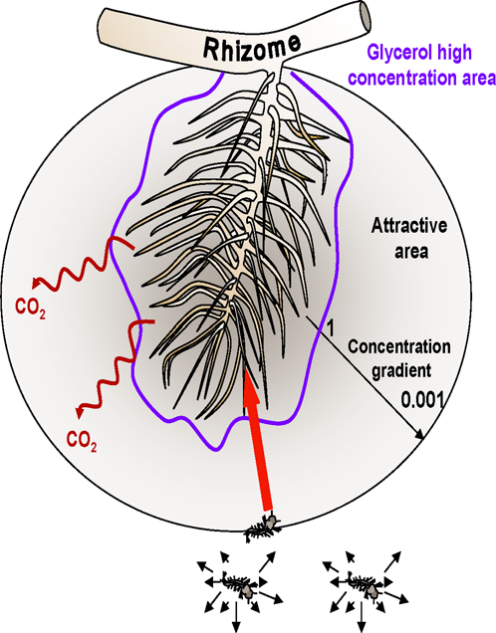
Hypothesis of the establishment of a root exudate gradient *in natura*.

*In natura*, the root exudates of *Typha latifolia* occur in a specific area characterized by the absence of water movement and the presence of high amounts of organic matter decomposing under anoxic conditions. Therefore, plant compounds released into this static environment form a concentration gradient by diffusion (Fig. 4). Because of the characteristics of this aquatic medium, it has been suggested that this concentration gradient could be quite stable locally. Associated with this zone, a specific bacterial flora may be present ensuring the decomposition of such compounds [32], [42], which could permit the stabilization of the root exudates gradient with time. Thus, *Coquillettidia* larvae in search of an oxygen source could have access to this specific area. Our studies demonstrated that root compound mixture diluted by a factor of 1000 was still attractive to these larvae. Moreover, Sérandour et al. [31] demonstrated that *Coquillettidia* larvae were positively attracted by CO_2_ emissions. Therefore, under natural conditions, the signal could be reinforced by the emanation of CO_2_ gas [32] from both plant roots and bacteria living near the rhizosphere [43]. We may postulate that the first step of larval attraction could be linked to a chemo-attraction signal composed of uracil, thymine, uridine, thymidine, and CO_2_. Larvae may be able to orientate their swimming in function of the type of compound (odor identity) and in function of the level of concentration (odour intensity) [44]. Therefore, because of a stagnant aquatic system and a continuous release of plant exudates, these attractants might constitute a very stable, long-term attractive signal. This behaviour may be relieved in a second step by a nutritional signal due to high concentrations of glycerol close to the roots, because of the probable low mobility of this compound in water. The rhizosphere is generally rich in sugars and bacteria, which are the principal source of food for the larvae [43], [45], [46]. Once the *Coquillettidia* larvae were in the proximity of the roots, they would be able to perforate suitable roots and thus reach the aerenchyme to aquire oxygen [31].

The elucidation of *Coquillettidia* larvae behaviour permitted to establish ecological parameters such as the glycerol-uracil-thymine-uridine-thymidine mixture attraction. *Coquillettidia* larvae are frequently associated with *T. latifolia* plants *in natura*. This could be explained by the fact that this plant is able to release an attractive mixture with an average rate of 9 nmol/day/plant, and has an extensive root system appropriate to the perforation by the larval siphon, which is not the case of *A. lanceolatum* and *G. fluitans*. Moreover, the extensive production of organic matter in the root surroundings could permit the presence of a diversity of micro-organisms which would be a source of nutrition for *Coquillettidia* larvae.

## Materials and Methods

### Plant materials

All plants were cultivated in controlled conditions as described below: 16:8-h light:dark; 70±5% RH; 25±2°C; 6500 lux.

Corn seeds (*Zea mays*) and sunflower (*Helianthus annuus*) plants were placed in pots containing soil and 10- to 14-d-old plants were used in all experiments.

Lanceleaved waterplantain (*Alisma lanceolatum*), floating sweet-grass (*Glyceria fluitans*) and broadleaf cattail (*Typha latifolia*) plants were collected *in natura* and maintained under controlled conditions in hydroponic Hoagland's solution. *Typha latifolia* plants were cultivated in these conditions and multiplied by cutting. New plants were transferred in glass containing Hoagland's nutritive solution with oxygenation.

### Insects

*Coquillettidia richardii* (Ficalbi) and *Coquillettidia buxtoni* (Edwards) larvae (4^th^ instar) were collected *in natura* (natural protected subalpine marsh wetland), observed for determination one by one and placed in clear water (pH 7) in the presence of *Typha latipholia* to allow adhesion and bacterial feeding. *Coquillettidia* cultures were maintained in dark chambers at 12±1°C and 70±2% RH.

Before each experiment, larvae were collected in clean water, in the presence of *T. latifolia* and put at room temperature under red light for 1 h. Larvae were used only once per day and no mortality was reported for the duration of the bioassay.

*Culex pipiens* larvae (4^th^ instar) collected in natural protected subalpine marsh wetlands and the laboratory strain, *Aedes aegypti* Bora-Bora, were reared in an insectary (26±2°C, 14:10h light:dark photoperiod, 80% RH and fed according to Chaton et al. [47]).

### Chemicals

Pure chemicals (amino acids, organic acids, sugars, purines, nucleosides, fatty acids, alcohols and flavonoids) were purchased from Sigma-Aldrich Co. (Lyon, France). Glycerol (RECTAPUR^TM^, 98% purity) was purchased from VWR International (Fontenay-sous-Bois, France). Hydroxamic acids (e.g. DIMBOA, 2,4-dihydroxy-7-methoxy-1,4-benzoxazin-3-one) were purified as described by Raveton et al. [48].

### Plant exudates collection

Plants (*Z. mays* and *T. latifolia*) were selected as a function of their healthiness and their stage of development. Plants (20 plants of 7-day-old for *Z. mays*; 5 plants of *T. latifolia*) were placed in sterilized tap water (V = 400 ml) in a container avoiding external contamination. Plants were incubated during a 1 d photoperiod of 16:8-h light:dark and conditions of 70±5% RH; 25±2°C; 6500 lux. Every 24 h, water-soluble root exudates were collected and filtered through Whatman No. 1 cellulose filter paper (Millipore, Saint-Quentin-en-Yvelines, France). Each day of collection, root exudates were directly tested on larvae attraction. For chemical analyses, a volume of 400 ml was lyophilized.

*In situ* samples were collected in sediments and after centrifugation of mud (15 min, 1 000*g*), water supernatants were withdrawn and tested directly for their attractiveness on *Coquillettidia*. Part of the water-sediment (700 ml) was filtered through Whatmann No. 1 and the filtrate was lyophilized for further chemical analyses.

### Chemical identification and quantification analyses

The lyophilized water soluble root exudates were solubilized in 50 µl of acetonitrile and 100 µl of BSTFA-TCMS reagent ((bis(trimethylsilyl)trifluoroacetamide/trimethylchlorosilane (99/1); Supelco, Sigma-Aldrich, Saint Quentin Fallavier, France). The reaction was carried out at 70°C for 20 min, followed by incubation at room temperature for at least 2 h. After centrifugation (10 min, 14 000*g*), the samples were ready for GC-MS analysis [49], [50].

GC-MS analysis was carried out on a HP6840/HP5973 apparatus (Agilent Technologies, Les Ulis, France) equipped with an MDN-12 fused silica capillary column (30 m, 0.25 mm internal diameter, 0.25 µm film; Supelco). The injector was used in the split mode, with a split ratio of 50∶1 and an injection volume of 2.5 µl. The oven temperature was held at 70°C for 4.5 min, then increased to 240°C at a rate of 50°C/min and held for a further 20 min (Injector temperature: 250°C; Detector temperature: 280°C). Glycerol, uracil, thymine, uridine and thymidine were used as external standards to identify exudate compounds by comparing their retention times and their mass spectra and referenced mass spectra from International Library (NIST/EPA/NIH Mass Spectral Library, Version 2.0d, 2005). These chemical references were used to standardize the SIM mode program (dwell time: 100 ms). The following SIM masses and retention times were used for disilylated-uracil (m/z: 241 and 256; t_R_: 7.60 min), disilylated-thymine (m/z: 255 and 270; t_R_: 7.79 min), tetrasilylated-uridine (m/z: 217 and 259; t_R_: 14.36 min) and trisilylated-thymidine (m/z: 171 and 261; t_R_: 13.72 min). GC-MS reference curves in duplicates (0.5, 1, 2.5, 5, 50, 500, 5000, 50000, 100000 µg/l) were established under the same conditions (silylation) for each standard: uracil (R^2^ = 0.99), thymine (R^2^ = 0.99), uridine (R^2^ = 0.99) and thymidine (R^2^ = 0.99).

Trisilylated-glycerol (t_R_: 7.33 min) was analyzed using the SCAN mode program (calibration curve (duplicates, R^2^ = 0.99): 1, 10, 50, 100, 1000 mg/l).

### Behaviourial bioassay

Plant roots (*Alisma lanceolatum*, *Glyceria fluitans*, *Typha latifolia, Zea mays*, *Helianthus annuus*) and compounds attraction screening was performed using a four-channel arena (see details in [31] and Fig. S1). The attraction of 20 larvae was evaluated under red light measuring by counting the number of larvae present in each channel every 5 min during the assay period (60 min). 10 replicates were performed for each condition. Behavioural response of *C. richiardii* and *C*. *buxtoni* larvae was similar (ANOVA, p>0.05) and they showed the same attraction response towards *T. latifolia*.

A two-choice olfactometer (see details in [51] and Fig. S2) was used to investigate the precise behavioural responses of *Coquillettidia sp.* larvae towards synthetic compounds. First, chemical compounds (glycerol, uracil, thymine, uridine, thymidine; solubilized in tap water filtered through Whatman No. 1 cellulose filter paper (Millipore, Saint-Quentin-en-Yvelines, France)) were tested individually at different concentrations to determine the attractive concentration. Next, mixtures of these chemicals mimicking maize (10 nM glycerol, 0.5 nM uracil, 0.08 nM thymine, 10 nM uridine, 120 nM thymidine) and cattail exudates (1 nM glycerol, 0.5 nM uracil, 0.04 nM thymine, 7 nM uridine, 110 nM thymidine) (Table 2) were tested with different dilution factors: 10, 0.1, 0.01, 0.001 and 0.0001. The olfactometer central arena (diameter = 10 cm; volume = 196 ml) and its 4-arms (length = 10 cm; volume = 50 ml) were filled with tap water. The solutions to be tested were conveyed *via* the 4-arms owing to a pump with a controlled flow fixed at 3.2 ml/min. Two juxtaposed arms received control solution (tap water) and the two other arms received the solution to be tested. At T0, one individual was placed in the center of the olfactometer for 3 min. A ‘no choice’ response was recorded when the larvae did not move or did not make a choice between the tested solutions. A ‘first choice’ response was noted when larvae moved inside one arm. All measurements were performed under red-light (650 nm) for which *Diptera* spp. are not sensitive [52].

### Statistical data analyses

For the screening of attraction, results were expressed as the average number of total larvae present in each channel for each measure time (means±SE). Statistical comparisons of the means were made using repeated-measures ANOVA using the SPSS 11.0 statistical program (SPSS Inc., Chicago, IL, USA).

Differences between the numbers of larvae choosing each arm of the olfactometer (First Choice in 3 min experiment) were analysed using a χ^2^ test, performed with the software Statview 4.57.0.0 for windows. No-choice results ranged from 1 to 15%, and were not significantly different between the control and test solution (ANOVA, P>0.05). Therefore, larvae that did not make a choice were eliminated from statistical analyses [22], [53]

## Supporting Information

Figure S1 Four-channel olfactometer for orientation bioassays of Coquillettidia larvae (top view). The preferential orientation of larvae towards a given plant or solution was investigated in the laboratory using 20 larvae per assay in a four-channel arena (34×22 cm). The larval preferential orientation towards a given plant or solution was evaluated by counting the number of larvae present in each channel every 5 min during the assay period (60 min). For each behavioural experiment, the plant or solution tested was put in a different channel to avoid a ‘channel effect’. Moreover, between each experiment, the arena was rinsed twice with ethanol and 10 times with tap water. For plant test, one living plant was placed at the end of one channel and tested against water in the three other channels. Only plant roots were immerged in water to test their attraction. For the solution test, the solutions to be tested were conveyed to the four-channels via a pump with a controlled flow fixed at 3.2 ml/min. One channel received the test solution and the three other arms received the control solution (tap water).
(0.14 MB TIF)

Figure S2 Two-choice olfactometer for attractiveness bioassays of Coquillettidia larvae. First choice experiments were carried in a two-choice oflactometer (adaptated from Saglio et al. [49]). The olfactometer central arena (diameter = 10 cm; volume = 196 ml) and its 4-arms (length = 10 cm; volume = 50 ml) were filled with tap water. The solutions to be tested were conveyed via the 4-arms owing to a pump with a controlled flow fixed at 3.2 ml/min. Two juxtaposed arms received control solution (tap water) and the two other arms received the solution to be tested. At T0, one individual was placed in the center of the olfactometer for 3 min. A ‘no choice’ response was recorded when the larvae did not move or did not make a choice between the tested solutions. A ‘first choice’ response was noted when larvae moved inside one arm. Between each experiment, the olfactometer was rinsed twice with ethanol and 10 times with tap water.
(0.24 MB TIF)
