# Supplementary figures and images for: Ubiquitous Water-Soluble Molecules in Aquatic Plant Exudates Determine Specific Insect Attraction
Source: PLoS One. 2008 Oct 8;3(10):e3350. doi: 10.1371/journal.pone.0003350 (PMC2556394; doi:10.1371/journal.pone.0003350)

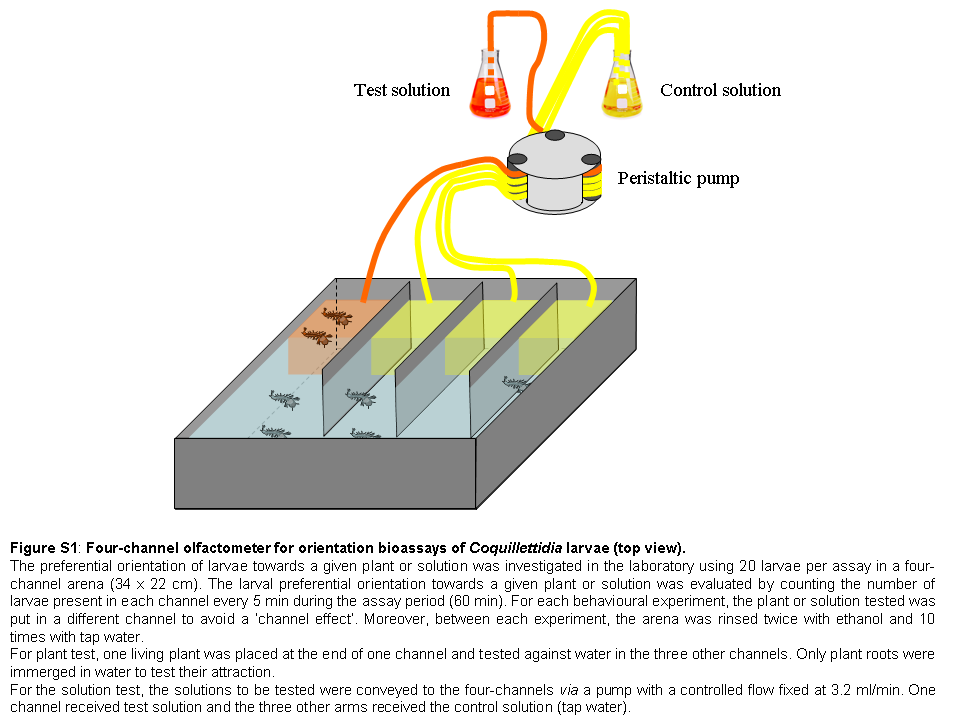

Supplement: Figure S1 — Four-channel olfactometer for orientation bioassays of Coquillettidia larvae (top view). The preferential orientation of larvae towards a given plant or solution was investigated in the laboratory using 20 larvae per assay in a four-channel arena (34×22 cm). The larval preferential orientation towards a given plant or solution was evaluated by counting the number of larvae present in each channel every 5 min during the assay period (60 min). For each behavioural experiment, the plant or solution tested was put in a different channel to avoid a ‘channel effect’. Moreover, between each experiment, the arena was rinsed twice with ethanol and 10 times with tap water. For plant test, one living plant was placed at the end of one channel and tested against water in the three other channels. Only plant roots were immerged in water to test their attraction. For the solution test, the solutions to be tested were conveyed to the four-channels via a pump with a controlled flow fixed at 3.2 ml/min. One channel received the test solution and the three other arms received the control solution (tap water). (0.14 MB TIF) [file pone.0003350.s001.tif]

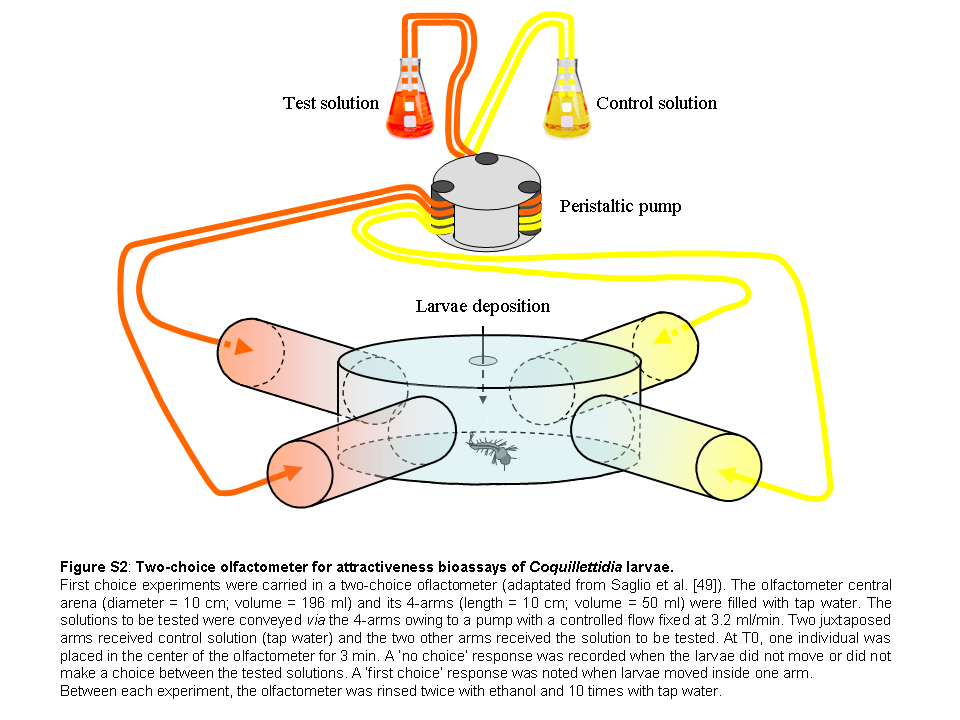

Supplement: Figure S2 — Two-choice olfactometer for attractiveness bioassays of Coquillettidia larvae. First choice experiments were carried in a two-choice oflactometer (adaptated from Saglio et al. [49]). The olfactometer central arena (diameter = 10 cm; volume = 196 ml) and its 4-arms (length = 10 cm; volume = 50 ml) were filled with tap water. The solutions to be tested were conveyed via the 4-arms owing to a pump with a controlled flow fixed at 3.2 ml/min. Two juxtaposed arms received control solution (tap water) and the two other arms received the solution to be tested. At T0, one individual was placed in the center of the olfactometer for 3 min. A ‘no choice’ response was recorded when the larvae did not move or did not make a choice between the tested solutions. A ‘first choice’ response was noted when larvae moved inside one arm. Between each experiment, the olfactometer was rinsed twice with ethanol and 10 times with tap water. (0.24 MB TIF) [file pone.0003350.s002.tif]
